# Loneliness among older adults in Europe: The relative importance of early and later life conditions

**DOI:** 10.1371/journal.pone.0267562

**Published:** 2022-05-18

**Authors:** Sophie Guthmuller

**Affiliations:** 1 Department of Socioeconomics, Health Economics and Policy group, Vienna University of Economics and Business, Vienna, Austria; 2 RWI Essen, Leibniz Science Campus Ruhr, Essen, Germany; University of Florence, ITALY

## Abstract

The aim of this paper is to study the association between childhood circumstances and loneliness in older adults in Europe. Based on rich information collected by the Survey on Health, Ageing, and Retirement in Europe (SHARE) on childhood characteristics and individual characteristics at age 50+, the study is able to control for personality traits, socioeconomic and demographic factors, social support and health in later life, and country-specific characteristics. The analyses show strong correlations between life circumstances in childhood and feeling lonely in older age; these correlations remain significant after adjusting for covariates. While ill health is the main factor correlated with loneliness at 50+, as expected, the analysis of the relative importance of the determinants reveals that personality traits account for more than 10% of the explained variance and that life circumstances during childhood account for 7%. Social support at older ages is the second highest category of factors, accounting for 27%—with, interestingly, support at home and social network characteristics contributing about 10% each, engaging in activities and computer skills accounting for 7% of the explained variance. Demographic and socioeconomic factors account for 6% and country-level characteristics contribute 5%. This paper points out the relevance of early life interventions to tackling loneliness in older age, and it shows that early interventions and interventions aiming at increasing social support in later life need to be adapted to all personality types. Thus, the role of childhood circumstances and the mechanisms explaining the association between loneliness in childhood and loneliness in later life deserve more attention in future research.

## Introduction

Loneliness has been a growing topic of interest in Europe over the last decade [1–4], as it has been shown to be linked with ill health and to increase with age. Loneliness is correlated with a higher risk of developing mental conditions (e.g., depression, dementia) and a deterioration in physical health (e.g., less active lifestyles, diabetes, stroke, coronary heart disease), as shown for instance in [5–11]. Loneliness is also linked with all causes of mortality [12–15] and has an impact on health care utilization [16]. In 2016, 6% of the European population declared that they felt lonely most of the time, and this proportion reached 9% for the population aged 65+ years [17].

In addition to ill health, which is one of the major predictors of loneliness among older adults, another set of individual characteristics in later life is known to be significantly correlated with loneliness. Among these characteristics are demographic and socioeconomic factors, household characteristics (e.g., size of household, marital status), and the amount of social support (participation in social activities, frequency of contact with family members and friends, size of social network, closeness to social network). For an overview, see, for instance [2, 18]. Recent studies have also shown that certain personality traits—in particular, neuroticism and extraversion–are significantly associated with loneliness [19–21].

However, there is less empirical evidence on the effect of life events on loneliness, especially of early life circumstances. The link between early life conditions and loneliness at age 50+ can be explained through several underlying mechanisms. The exposure to stressful events in childhood (e.g. financial distress, ill health, physical harm) or the child social environment (e.g. the parent–child relationship, friendships) might influence its social development and self-esteem, which could have direct and indirect long-lasting effects on the child’s risk of suffering from loneliness later in life. Numerous studies have found significant effects of early life conditions on later life outcomes, including health-related variables, educational attainment, and employment [22–30].

The majority of the studies that examined the determinants of loneliness among older adults are cross-sectional [31–34] with few exceptions, to the best of our knowledge. For instance, the paper by Aartsen and Jylhä analyzed the onset of loneliness based on a prospective study in Tampere, Finland; they found that life events such as losing a partner have a significant effect on loneliness [35]. Using national survey data in Sweden, Dahlberg et al. studied the link between social engagements in early life and social engagement in later life and how it is correlated with feelings of loneliness [36]. The only study that focused on the link between early life circumstances and loneliness in later life is the one by Kamiya et al. [37]. The study showed that poor childhood socioeconomic status and parental substance abuse have direct effects on loneliness at older ages in Ireland, after accounting for demographic, socioeconomic, and health factors.

This paper aims to contribute to the existing literature by answering the following questions. First, are life circumstances in childhood significantly associated with loneliness in later life in Europe? If the answer is yes, does the significant association remain after controlling for personality traits, demographic and socioeconomic factors, social support and health in later life, and country-specific fixed effects? Second, what is the relative importance of the link between childhood circumstances and loneliness in later life, compared to later life conditions, including country-specific characteristics?

This study addresses these questions using rich individual-level data from 17 countries in Europe allowing accounting for a larger set of individual characteristics in early life and later life, and in particular, country specific fixed effects. Loneliness is measured with the R-UCLA Loneliness Scale. It uses indirect questions that do not mention the word “loneliness” in order to account for under-reporting related to the social stigma of suffering from loneliness [38–40].

The remainder of the paper is structured as follows. Section “Background” presents the evidence from the empirical literature and the underlying mechanisms that explain the link between early life circumstances and loneliness at older ages. Section “Subjects and methods” describes the data and the estimation strategy. The results are reported in Section “Results”. Section “Discussion” discusses the results and limitations of the study.

## Background

Loneliness is defined as “a situation experienced by the individual as one where there is an unpleasant or inadmissible lack of (quality of) certain relationships. This includes situations in which the number of existing relationships is smaller than is considered desirable or admissible, as well as situations where the intimacy one wishes for has not been realized” [18]. Therefore, loneliness is a multidimensional phenomenon that may include (subjective) social isolation—the (perceived) “lack of, or deficit in, the quantity of a social network” [41], and emotional isolation—“the lack of person(s) to whom one feels attached” [41].

While social isolation and loneliness are strongly associated, lonely persons are not necessarily socially isolated, and persons with a small social network do not necessarily feel lonely. Hence, loneliness is a subjective and complex feeling moderated by different individual factors that can be grouped as follows: childhood circumstances, personality traits, demographic and socioeconomic factors, social support and health, and macro-level characteristics. The remainder of this section “Background” presents the evidence from the literature and describes the underlying mechanism through which these factors could predict later life loneliness.

### Childhood circumstances

The mechanisms through which early social relationships can be associated with loneliness in later life are mainly grounded in social developmental theories [42]. Based on Bowlby and Ainsworth’s theory of attachment [43–45], it has been shown that the parent–child relationship—and the mother–child relationship in particular—plays a crucial role in developing secure attachment skills, leading to better social, emotional, and cognitive skills later in life [46, 47]. A lack of (close) friendships is similarly detrimental to the development of social skills, self-esteem, and self-perception, and it leads to increased long-lasting feelings of rejection [48, 49]. The role of friendship has been significantly linked to later life outcomes related to social development and cognitive functioning, as well as to mental health and well-being [50–53]. More generally, a number of empirical studies have shown the importance of the link between early social relationships and outcomes in later life. For instance, Ejlskov et al. showed that relationship adversities throughout life, including in childhood, increase the risk of feeling lonely in later life [54]. Previous research also revealed that socially isolated children tend to have lower subsequent educational attainment and be part of a less advantaged social class in adulthood, and that they are more likely to be psychologically distressed in adulthood [55].

The link between early life conditions and loneliness at age 50+ can be explained by exposure to stressful events in childhood, such as financial distress, ill health, and physical harm, which have been shown to have long-lasting effects on a child’s well-being and various later life outcomes; health-related variables, educational attainment, and employment [22–30]. Early life conditions may have a direct link to loneliness in later life through personal constraints (deficit in social skills, low self-esteem, powerlessness, expectations about self-efficacy, self-perceived lack of disclosure to others) [18, 56] that remain over the course of life. They may also have an indirect link—for instance, through educational attainment, employment status, or relationship status—that in turn affect loneliness at age 50+. A couple of studies examined the correlation between early life conditions and risk of loneliness in later life [37, 57]. Kamiya et al. found that parental substance abuse and financial distress during childhood are associated with an increased level of loneliness in later life [37]. Childhood trauma (and adulthood trauma) were independently related to the most distressed loneliness classes [58].

The importance of religion in the family is generally linked with a set of norms and cultural constraints that favor collectivistic values and moderate self-definition and role conception of individuals within the group. In this view, growing up in a family with strong collective values influences self-perception on being alone, feeling lonely, and social connectedness [59]. It has been shown that, in collective societies, individuals with fewer social connections tend to feel lonelier, as they do not comply with a cultural value in which the focus is the group. They therefore tend to feel more responsible for their lack of social network and their feeling of loneliness [60, 61]. In more individualistic societies, individuals tend to be more independent, oriented to themselves, and thus might be less impacted by fewer contacts or social isolation.

### Personality traits

Recent research shows that the propensity of feeling lonely is also linked to personality. Neurotic and anxious personalities are expected to be positively linked to loneliness, whereas extraversion, agreeableness, and conscientiousness tend to be protective personality traits [62]. Using the five big personality traits, Wang et al. found that neuroticism was significantly correlated with a higher level of loneliness in older adults [20]. In a longitudinal study following subjects from adolescence to midlife, neuroticism was found to predict levels of subjective health and loneliness later in life [63]. Extraverted older adults, on the other hand, were found to suffer less from loneliness [21, 64]. using multivariate models controlling for individual confounding factors in adulthood, Buecker et al. found that all personality traits were significantly associated with loneliness, except openness [65]. Neuroticism was found to predict the development of subjective health and loneliness later in life.

### Demographic and socioeconomic factors

The literature has shown that loneliness varies across age groups and life stages. Among older adults, one would expect that loneliness increases with age and the likelihood of being alone. With a higher life expectancy, women are also more exposed to loneliness than men. However, the evidence from the literature regarding these two factors of age and gender are rather mixed, indicating that other associated factors, such as health and social support, might have a larger impact on loneliness [66–69].

Another factor associated with age and gender relates to work situation and wealth. Having employment is expected to be linked with more frequent social interactions and higher socioeconomic status, which in turn leads to a lower risk of loneliness. Empirical results indeed found that the retired and unemployed populations have a larger likelihood of suffering from loneliness. The higher risk of loneliness among those with low levels of income can be partly explained by their more frequent experience of stressful situations in order to be able to make ends meet, and less frequent participation in unaffordable social activities. See, for instance, [70, 71].

### Social support in later life

Social support is one of main moderators of loneliness in later life. Comparing the onset of loneliness in different countries, Sundström et al. found that living alone was the most consistent factor related to a higher level of loneliness [33]. Adverse family life events among older adults have also been found to be largely associated with loneliness. Vozikaki et al. showed that a child’s departure from home or the death of a partner are major predictors of steady feelings of loneliness [31]. Beyond family characteristics, another part of the literature focuses on the link between social connection and loneliness in older adults. Several papers in Europe and in the US showed that having a network of confidants has a greater effect in preventing loneliness than living alone [10, 34, 72–74]. In addition to regular contact with family and friends, taking part in social activities is also a protective factor of loneliness. Niedzwiedz et al. even found that social participation reduces the socioeconomic differences in loneliness among older adults in Europe [70]. The environment, such as living in a rural area or in a large city, is associated with higher levels of loneliness, although living in a deprived region matters more [75–77].

Access to technology and the internet, in particular social media, has also been linked to mental health issues and loneliness, especially among young adults and adolescents [78]. Among older adults, internet communication and social media tools are used primarily to stay in contact with children, other family members, and friends. As such, they are associated with a lower level of loneliness [79].

### Health conditions in later life

Ill health is among the main predictors of social isolation and is therefore significantly correlated with loneliness. Older adults with ill health are more likely to suffer from loneliness—see, for instance, Barlow et al. and Meltzer et al. who studied levels of loneliness in chronically ill persons or adults with mental disorders [80, 81]. Health conditions—such as chronic diseases, IALD limitations, depressive symptoms, and subjective ill health status—are found to be strong risk factors for social isolation and loneliness [31, 33, 82].

### Country-specific characteristics

In addition to these individual characteristics, country-specific characteristics also explain higher risks of loneliness. Macro-level demographic and economic factors, inequalities, cultural norms and values, levels of safety, and the existence and extent of public and social policies are among the country-specific characteristics that were found to be associated with social isolation and loneliness [32, 33, 83–85].

## Subjects and methods

### Survey on Health, Ageing, and Retirement in Europe (SHARE)

The data from the Survey on Health, Ageing, and Retirement in Europe (SHARE) provide the information for individuals aged 50+ on health, socioeconomic status, and social and family networks. The cross-national panel database covers 27 European countries and Israel. The regular questionnaire (SHARE) has been carried out every two years since 2004. In addition to the regular questionnaire, a SHARELIFE questionnaire was carried out in 2009 (wave 3) and in 2017 (wave 7). The SHARELIFE questionnaire is a retrospective survey collecting information about past and early life experiences related to employment, health, family, and housing situation. The questionnaire followed a so-called life history calendar (LHC), helping respondents remember the chronology of past events. The individuals included in the study are those aged 50+ who participated in wave 6 [86] and replied to the SHARELIFE history questionnaire in wave 7 [87, 88]. The individuals live in Austria, Germany, Sweden, Spain, Italy, France, Denmark, Greece, Switzerland, Belgium, Israel, the Czech Republic, Luxembourg, Portugal, Slovenia, Estonia, and Croatia.

#### Loneliness at 50+

The data to characterize individuals at 50+ come from wave 6 (2015). The short version of the Revised UCLA Loneliness Scale [38–40, 89] is used as the outcome of interest. This scale is based on the answers to three items: How much time do you feel…: (i) you lack companionship; (ii) left out; (iii) isolated from others? Participants responded to these three items on a three-point Likert scale: “often” (3), “some of the time” (2), “hardly ever or never” (1). The addition of points from the three items determines the R-UCLA Loneliness, which ranges from 3 (not lonely) to 9 (very lonely). This paper studies the determinants of moderate to severe levels of loneliness, taking into account the differences in self-assessed loneliness across countries. Therefore, respondents are defined with a level of loneliness scale, in which the fourth country-specific quartile was defined as “feeling lonely” and those in the first, second, and third quartiles were defined as “not lonely.” Focusing on the highest quartile of the lonely population is most relevant for policy implications. From a statistical point of view, this definition is also used to account for the non-normal distribution of the loneliness scale among respondents [70, 90].

#### Childhood circumstances

Information on childhood (before age 17) is retrieved from the wave 7 SHARELIFE questionnaire. Participants were asked to rank how they felt during childhood about a number of items. For childhood circumstances, the following items were considered: having a group of friends they felt comfortable spending time with; their relationship with their mother/father; whether their family was pretty well off financially, about average, or poor; their subjective health status; and the importance of religion at home. A dummy variable was also added to indicate whether the child was the only child in the household. Finally, the variable “never being physical harmed” was recoded as (never vs. often, sometimes, rarely) from the question “How often did your mother / father / or another person that raised you, push, grab, shove, throw something at you, slap or hit you?”

#### Personality traits

The 10-item Big Five Inventory (BFI-10) is used to study the association between personality and feelings of loneliness, introduced for the first time in wave 7. It is an established personality inventory measuring the “Big Five” personality dimensions with two items each. Introduced by Rammstedt and John in 2007 [91, 92] the BFI-10 is an ultra-short measure of personality, which is especially suitable for multi-theme surveys in which assessment time and questionnaire space are limited. As such, the BFI-10 measures the following five personality traits: openness vs. closedness to experience; conscientiousness vs. lack of direction; extraversion vs. introversion; agreeableness vs. antagonism; and neuroticism vs. emotional stability. Each personality trait has a score ranging from low to high, as 1(0.5)5 [91, 92].

#### Demographic and socioeconomic characteristics

As previously shown in the literature, differences in gender, age, and highest education level (divided into low, or medium and high), and employment status are controlled for as drivers of hampering factors of loneliness. Wealth, as an economic resource variable equalized by the OECD equivalence scale, is used and defined as country-specific quintiles to account for the difference in employment status. Wealth includes household financial (income, money in bank accounts, etc.) and real assets (value of own residence or vehicle).

#### Social support at 50+

To measure social support at home, the marital status and size of the household are included. Marital status is defined as being married or in a civil partnership, compared to divorced/separated, never married, or widowed. The size of the household considers the number of individuals (one, two, three, or more). The set of variables available in the module dedicated to social network in wave 6 is used to measure the quality of the social support received inside and outside of the family. The social network is defined as persons with whom one most often talks about important things—the person can be a family member, friend, neighbor, acquaintance, etc.). The size of the network (the number of persons in the social network), the frequency of contact with the social network (daily, several times a week, about once a week, less than once a week to never), the mean closeness of the social network (defined as a categorical variable with three levels: close, very close, extremely close), and the geographical proximity to the social network (same household, less than 1 kilometer away, between 1 and 25 kilometers away, more than 25 kilometers away) are included. The geographical environment is identified by proxy through information on the area where the household is located (a rural area or village, a big city, the suburbs or outskirts of a big city, a large town, a small town).

To measure social participation, information on participation in activities and self-assessed computer skills are used. The variables of number of activities the last twelve months and level of satisfaction with activities were combined to create a categorical variable that takes into account the level of satisfaction of individuals reporting participation in any activity. Two subgroups are defined of those who replied that they have participated in any activity: those who are satisfied with no activity (rated 5 or more on a scale of 1 to 10) and those who are not satisfied with no activity (rated 4 or less). The number of activities is then composed of the following items: none & unsatisfied; none & satisfied; one, two, three, four & more. The variable of computer skills consists of the following categories: excellent or very good, good, fair, poor, I never used a computer.

#### Health status at 50+

To control for ill health status as a major loneliness driver, the following are used: information on the number of chronic diseases (none, one, two or more); the EURO-D scale that measures depression; and the Global Activity Limitation Index (GALI), which defines ill health as “being limited” for six months or more in activities people usually do [93].

#### Sample

The working sample has the following main characteristics. It includes 27,623 observations for which there were no missing values in the variables used in the analysis. The initial sample includes 33,523 observations (See S1 Appendix for more detailed information on the exclusion criteria). The analysis of the missing observations by variable shows that the proportion of missing observations ranges from 0.04% to 6.06%. The analyses were run including a missing category when applicable, and the estimates and the findings do not vary with and without the inclusion of the missing observations. In addition, a very small proportion of some variables (maximum 1%) are imputed values—except for income, for which the proportion of imputed values is around 30%. Multiple imputation techniques (hot-deck method and fully conditional specification method) have been used by the SHARE project data team. (See detailed description of the methods and the procedures in [94] and [87]. A sensitivity analysis was performed to assess whether the results are affected by the imputed values, and it concludes that the estimates are robust. The results are available upon request.

The working sample is 57.10% female, with an average age of 67 years old; 57.94% are retired, and 71.53% are married. 37.31% have a low educational level. The most frequent household size is two individuals (57.45%), and 19.56% are single households. 35% live in a rural area. Regarding loneliness, the average loneliness scale is 3.85 (min = 3, max = 9), and 17.11% of individuals have a loneliness score within the fourth country-specific quartile. The figures are reported in Tables 1–4.

**Table 1:** Loneliness status at age 50+ by childhood circumstances and personality traits.

LONELINESS	Feeling lonely at age 50+					
CHILDHOOD CHARACTERISTICS	Total	Yes	No	Diff	s.e.	Chi2
**Friends with whom they were comfortable spending time**						**239.23**
Often	0.6923	0.603	0.7107	-0.108***	0.0114	
Sometimes	0.1891	0.2258	0.1815	0.044***	0.0081	
Rarely or never	0.1186	0.1712	0.1078	0.063***	0.0093	
**Relationship with mother**						**229.22**
No living mother	0.0046	0.0059	0.0043	0.002	0.0009	
Excellent	0.3017	0.2601	0.3103	-0.050***	0.0098	
Very good	0.3295	0.2948	0.3367	-0.042***	0.0102	
Good	0.2627	0.2836	0.2584	0.025*	0.0094	
Fair/Poor	0.1015	0.1556	0.0904	0.065***	0.0114	
**Relationship with father**						**148.47**
No living father	0.0275	0.0328	0.0264	0.006*	0.0024	
Excellent	0.2319	0.2078	0.2368	-0.029***	0.0068	
Very good	0.2946	0.2516	0.3034	-0.052***	0.0085	
Good	0.2948	0.3077	0.2921	0.016	0.0079	
Fair/Poor	0.1513	0.2	0.1412	0.059***	0.0099	
**No other child in household**	0.2567	0.3039	0.247	0.057**	0.0159	
**Physical harm**	0.5825	0.6277	0.5732	0.055***	0.0109	
**Bad health**	0.3778	0.4533	0.3622	0.091***	0.0124	
**Wealth**						**189.13**
Pretty well off financially	0.1176	0.1041	0.1204	-0.016*	0.0064	
About average	0.6211	0.5556	0.6346	-0.079***	0.0085	
Poor	0.2374	0.3134	0.2218	0.092***	0.0107	
Other	0.0238	0.0269	0.0232	0.004	0.0031	
**Religion**						**81.89**
Very important	0.2645	0.3115	0.2547	0.057***	0.013	
Somewhat important	0.3169	0.3166	0.317	0	0.0142	
Not very important	0.2297	0.2129	0.2331	-0.020*	0.0086	
Not at all important	0.1889	0.1589	0.1951	-0.036	0.0177	
PERSONALITY TRAITS						
Extraversion	3.7059	3.6618	3.7150	-0.053*	0.0184	
Agreeableness	4.1350	4.0485	4.1528	-0.104***	0.019	
Conscientiousness	3.5219	3.3184	3.5639	-0.245***	0.0211	
Neuroticism	2.6260	2.9663	2.5558	0.411***	0.0366	
Openness to experience	3.3274	3.2263	3.3483	-0.122**	0.0306	
**N =**	**27,623**	**4,725**	**22,898**			

Note: Feeling lonely at age 50+ is equal to one when respondents reported a loneliness score in the fourth country-specific quartile. Reading example: 60.3% of the lonely population reported having often friends with whom they were comfortable spending time. Differences in mean and clustered standard errors at the country level (s.e.) are displayed.

* p < 0.05,

** p < 0.01,

*** p < 0.001.

Chi2 is also reported for categorical variables.

**Table 2:** Loneliness at age 50+ by demographic, socioeconomic, and health factors.

LONELINESS	Feeling lonely at 50+					
	Total	Yes	No	Diff	s.e.	Chi2
**Loneliness scale**	3.8553	6.1630	3.3792			
DEMOGRAPHIC FACTORS						
**Female**	0.5710	0.6512	0.5545	0.097***	0.0117	
**Age**	66.6892	68.2159	66.3742	1.842***	0.3335	
SOCIOECONOMIC FACTORS						
**Employment status**						343.06
Employed	0.2723	0.1801	0.2913	-0.111***	0.0102	
Retired	0.5794	0.6076	0.5736	0.034*	0.0129	
Other	0.1483	0.2123	0.1351	0.077***	0.0099	
**Low education**	0.3731	0.4853	0.3500	-0.135***	0.0144	
**Income quintiles**						590.33
1st quintile	0.1920	0.2838	0.1730	0.111***	0.0118	
2nd quintile	0.1985	0.2519	0.1875	0.064***	0.0108	
3rd quintile	0.2017	0.1879	0.2045	-0.017	0.0084	
4th quintile	0.2060	0.1539	0.2167	-0.063***	0.0065	
5th quintile	0.2019	0.1225	0.2182	-0.096***	0.0054	
HEALTH						
**Chronic disease**						459.90
None	0.2375	0.1477	0.2560	-0.108***	0.0086	
One	0.2936	0.2474	0.3032	-0.056***	0.0093	
More than 1	0.4688	0.6049	0.4408	0.164***	0.0104	
**EURO-D caseness**	0.2528	0.5465	0.1922	0.354***	0.0166	
**Limitations with daily activities**	0.4494	0.6301	0.4121	0.218***	0.0075	
**N =**	**27,623**	**4,725**	**22,898**			

Note: Feeling lonely at age 50+ is equal to one when respondents reported a loneliness score in the fourth country-specific quartile. Reading example: 25.60% of the non-lonely do not have any chronic disease. Differences in mean and clustered standard errors at the country level (s.e.) are displayed.

* p < 0.05,

** p < 0.01,

*** p < 0.001.

Chi2 is also reported for categorical variables.

**Table 3:** Loneliness and social support at age 50+.

LONELINESS	Feeling lonely at 50+					
SOCIAL SUPPORT	Total	Yes	No	Diff	s.e.	Chi2
**Marital status**						657.42
Married	0.7153	0.5663	0.7460	-0.180***	0.0165	
Divorced	0.1012	0.1374	0.0938	0.044***	0.0085	
Never married	0.0509	0.0734	0.0463	0.027***	0.0045	
Widowed	0.1326	0.2229	0.1139	0.109***	0.0137	
**Size of the household**						695.59
One	0.1956	0.3342	0.1670	0.167***	0.0144	
Two	0.5745	0.4785	0.5943	-0.116***	0.0147	
Three and more	0.2299	0.1873	0.2387	-0.051***	0.0097	
**Size of the network**	2.7092	2.5498	2.742	-0.192***	0.0447	
**Frequency of contact with the network**						168.73
Daily	0.4271	0.3750	0.4378	-0.063***	0.0115	
Several times a week	0.3516	0.3412	0.3537	-0.013	0.0126	
About once a week	0.1633	0.1943	0.1570	0.037**	0.0107	
Less than once a week to never	0.0580	0.0895	0.0515	0.038***	0.0047	
**Closeness with the network**						285.50
Close	0.0644	0.1086	0.0553	0.053***	0.0063	
Very close	0.4604	0.5031	0.4517	0.051**	0.015	
Extremely close	0.4751	0.3884	0.4931	-0.105***	0.0138	
**Proximity to the network**						212.89
In same household	0.2317	0.1812	0.2421	-0.061***	0.0118	
Less than 1 km away	0.3088	0.2889	0.3129	-0.024*	0.0113	
Between 1 and 25 kms away	0.3812	0.4091	0.3754	0.034*	0.0156	
More than 25 kms away	0.0784	0.1208	0.0696	0.051***	0.0085	
**Area of living**						14.86
A big city	0.1573	0.1446	0.1599	-0.015	0.0158	
The suburbs of a big city	0.1005	0.1117	0.0982	0.014*	0.0063	
A large town	0.1292	0.1348	0.1281	0.007	0.0072	
A small town	0.2641	0.2597	0.2650	-0.005	0.0123	
A rural area or village	0.3489	0.3492	0.3488	0	0.0178	
** N =**	**27,623**	**4,725**	**22,898**			

Note: Feeling lonely at age 50+ is equal to one when respondents reported a loneliness score in the fourth country-specific quartile. Reading example: 14.46% of lonely individuals live in a big city. Differences in mean and clustered standard errors at the country level (s.e.) are displayed.

* p < 0.05,

** p < 0.01,

*** p < 0.001.

Chi2 is also reported for categorical variables.

**Table 4:** Loneliness and social support at age 50+.

LONELINESS	Feeling lonely at 50+					
SOCIAL SUPPORT	Total	Yes	No	Diff	s.e.	Chi2
**Number of activities**						524.81
None & unsatisfied	0.0568	0.1187	0.0440	0.075***	0.0173	
None & satisfied	0.0841	0.0876	0.0834	0.004	0.0061	
1	0.2207	0.2453	0.2156	0.030*	0.0133	
2	0.2456	0.2419	0.2464	-0.004	0.0134	
3	0.2015	0.1733	0.2073	-0.034***	0.0062	
4 and more	0.1913	0.1331	0.2032	-0.070***	0.0107	
**Computer skills**						428.03
Excellent or very good	0.1266	0.0762	0.1370	-0.061***	0.0081	
Good	0.2044	0.1543	0.2147	-0.060***	0.0061	
Fair	0.2304	0.2080	0.2350	-0.027*	0.0104	
Poor	0.1465	0.1630	0.1431	0.020*	0.0087	
I never used a computer	0.2921	0.3985	0.2702	0.128***	0.0176	
**N =**	**27,623**	**4,725**	**22,898**			

Note: Feeling lonely at age 50+ is equal to one when respondents reported a loneliness score in the fourth country-specific quartile. Reading example: 12.66% of the sample reported having excellent or very good computer skills. Differences in mean and clustered standard errors at the country level (s.e.) are displayed.

* p < 0.05,

** p < 0.01,

*** p < 0.001.

Chi2 is also reported for categorical variables.

### Statistical analysis

#### Descriptive analysis

A descriptive analysis is performed to compare the individual characteristics of lonely vs. not lonely individuals. Tests of difference in mean between the two groups are used for age and the UCLA loneliness scale. Tests of difference in proportion between the two groups are used for binary variables and for each item of the categorical variables. Standard errors are adjusted for clustering at the country level. The Pearson Chi2 test of independence is also reported for the categorical variables.

#### Multivariate equation model

The equation defining the relationship between loneliness at age 50+ and its determinants can be written as follows:

$$L_{i}=\beta_{0}+\beta_{1}C_{i}+\beta_{2}P_{i}+\beta_{3}D_{i}+\beta_{4}S_{i}+\beta_{5}H_{i}+\beta_{6}I_{c}+e_{i}$$ (1)

*L*_*i*_ is a dummy variable indicating whether an individual *i* has a level of loneliness at its country-specific fourth quartile of the R-UCLA scale. *C*_*i*_ is a vector of childhood circumstances (wealth; health; quality of relationship with mother, father, and friends; importance of religion). *P*_*i*_ is a vector of the five scores of the personality traits (extraversion, agreeableness, conscientiousness, neuroticism, and openness). The vector *D*_*i*_ includes a set of individual socioeconomic and demographic variables at age 50+. *S*_*i*_ is composed of social support, activities, and network variables at age 50+. *H*_*i*_ is a vector of individual health information. *I*_*c*_ is a country-specific fixed effect to account for differences in loneliness level between countries. *e*_*i*_ is the error term accounting for unobserved characteristics. Eq 1 is estimated with a logit model and clustered standard errors at the country level. Five specifications of the model were estimated, including each category of factors consecutively. Specification 1 includes the vector *C*_*i*_. Specification 2 includes the vectors *C*_*i*_ and *P*_*i*_. Specification 3 includes *C*_*i*_, *P*_*i*_, and *D*_*i*_. Specification 4 includes *C*_*i*_, *P*_*i*_, *D*_*i*_, and *S*_*i*_. Specification 5 is the full specification and includes *C*_*i*_, *P*_*i*_, *D*_*i*_, *S*_*i*_, and *H*_*i*_. All five specifications are estimated with country fixed effects (*I*_*c*_).

The variables included in the model were selected following a stepwise (forward hierarchical selection by category of factors, and backward selection within category) procedure and the Akaike Information Criteria (AIC). The pseudo R^2^ and the proportion of correctly specified outcomes are reported as measures of model fit for each of the specifications. Potential multicollinearity issues were ruled out using the correlation matrix and Variance Inflation Factor (VIF). Results available upon request.

#### Decomposition of the effects

Once the influence of each characteristic on the probability of feeling lonely is known, an analysis of the importance of the association between different group of factors (e.g., life circumstances in childhood, social support in later life) and loneliness in later life can be conducted. To measure the relative importance of each category of factors, the relative contribution of each these categories within the explained variance of Eq 1 is computed [95]. The computation follows McKelvey and Zavoina in 1975 [23, 96], breaking down the pseudo *R*^2^ to measure the share of variance explained by each category of variables *X*^*c*^ associated with a coefficient *β*^*c*^, using linear predictions of loneliness. The ratio (*R*_*c*_) of the contribution of each category of variable c is computed as follows:

$$R_{c}=\frac{cov\left(\hat{L^{*}},\beta^{c}X^{c}\right)}{Var\left(\hat{L^{*}}\right)}$$ (2)

where $\hat{L^{*}}$ is the linear prediction of Eq 1. Standard errors of the ratios are bootstrapped iterations.

Overall, the following p-value thresholds are reported in the statistical analysis; * p < 0.05, ** p < 0.01, *** p < 0.001. Given the sample size, results with p-values below 0.01 are commented as statistically significant in the text.

## Results

### Descriptive analysis

Among the population reporting feeling lonely at age 50+, the proportion of individuals reporting often having friends with whom they felt comfortable spending time with during childhood is significantly lower compared to the population without feelings of loneliness at age 50+. Similarly, the frequency of growing up with no other child in the household is 5.7 percentage points higher among individuals feeling lonely at age 50+. The quality of the relationship with parents is also negatively correlated with loneliness in older age. The proportions of individuals with excellent or very good relationships with their mother or father are significantly lower, while the proportions of those reporting a fair or poor relationship are higher among the subsample of lonely individuals aged 50+. This pattern is also found when looking at health and wealth indicators; the proportions of having experienced physical harm, having bad health, and growing up in a poor household are all significantly higher among the lonely subgroup at age 50+. Finally, individuals growing up in a household in which religion was very important are also more frequent among the lonely population in older age (see Table 1).

The two populations also differ by their personality traits. The score for agreeableness, conscientiousness, and openness are significantly lower within the lonely population, while the score for neuroticism is significantly higher (see Table 1).

Regarding the demographic and socioeconomic factors, the proportion of women is 9.7 percentage points higher among those feeling lonely compared to those without feelings of loneliness. Lonely individuals at age 50+ are around two years older. The proportion of employed individuals is lower among lonely individuals, while the proportion of “other types of employment” status is higher among the lonely population. This category includes the unemployed, homemakers, self-employed, and permanently sick individuals. The proportions of individuals with low educational level and lower income (1st and 2nd quintiles) are also higher among individuals feeling lonely. Conversely, the proportion of individuals with higher income (4th and 5th quintiles) is higher among the population without feelings of loneliness (see Table 2).

In terms of health, individuals with more than one chronic disease, limitations on activities, and symptoms of depression are significantly more common among the population feeling lonely at age 50+ (see Table 2).

Table 3 reports the quantity and quality of social connection by loneliness status at age 50+. As expected, the two subgroups differ in their social connections. First, social connections within the household are found to be protective factors for loneliness. The proportion of married individuals is 18.0 percentage points lower among the lonely population. The proportions of widowed, never married, and divorced individuals are higher within the lonely population. Similarly, one-person households are more frequent among the lonely group.

Second, the size and the tightness of the social network also seem to play an important role in impeding feelings of loneliness. The average size of the network—the number of persons with whom one can discuss important things is slightly lower among lonely individuals (around 2.5 vs. 2.7). The level of closeness with the network, frequency of contact, and proximity to the social network, are significantly different between the populations with and without feeling of loneliness. The proportion of the sample with daily contact with the network is on average 6.3 percentage points lower within the group of lonely individuals. The proportion of the sample reporting feeling extremely close to their network is 10.5 percentage points lower among the lonely population. The average geographical proximity to the persons within the network is also higher for individuals feeling lonely. The area of living, however, does not seem to differ between the two groups.

Third, the loneliness status differs with the engagement in activities and the level satisfaction. The proportion of individuals reporting participating in no activities and being unsatisfied is 7.5 percentage points higher in the lonely group. The proportion of those engaging in more than two social activities are also significantly lower in the population feeling lonely at age 50+. Interestingly, the proportion of those with at least a good level of computer skills is significantly lower in the lonely population; while the proportion of individuals reporting having never used a computer is 12.8 percentage points higher within the lonely group (see Table 4).

### Multivariate analysis

#### Childhood circumstances

**Fig 1:**
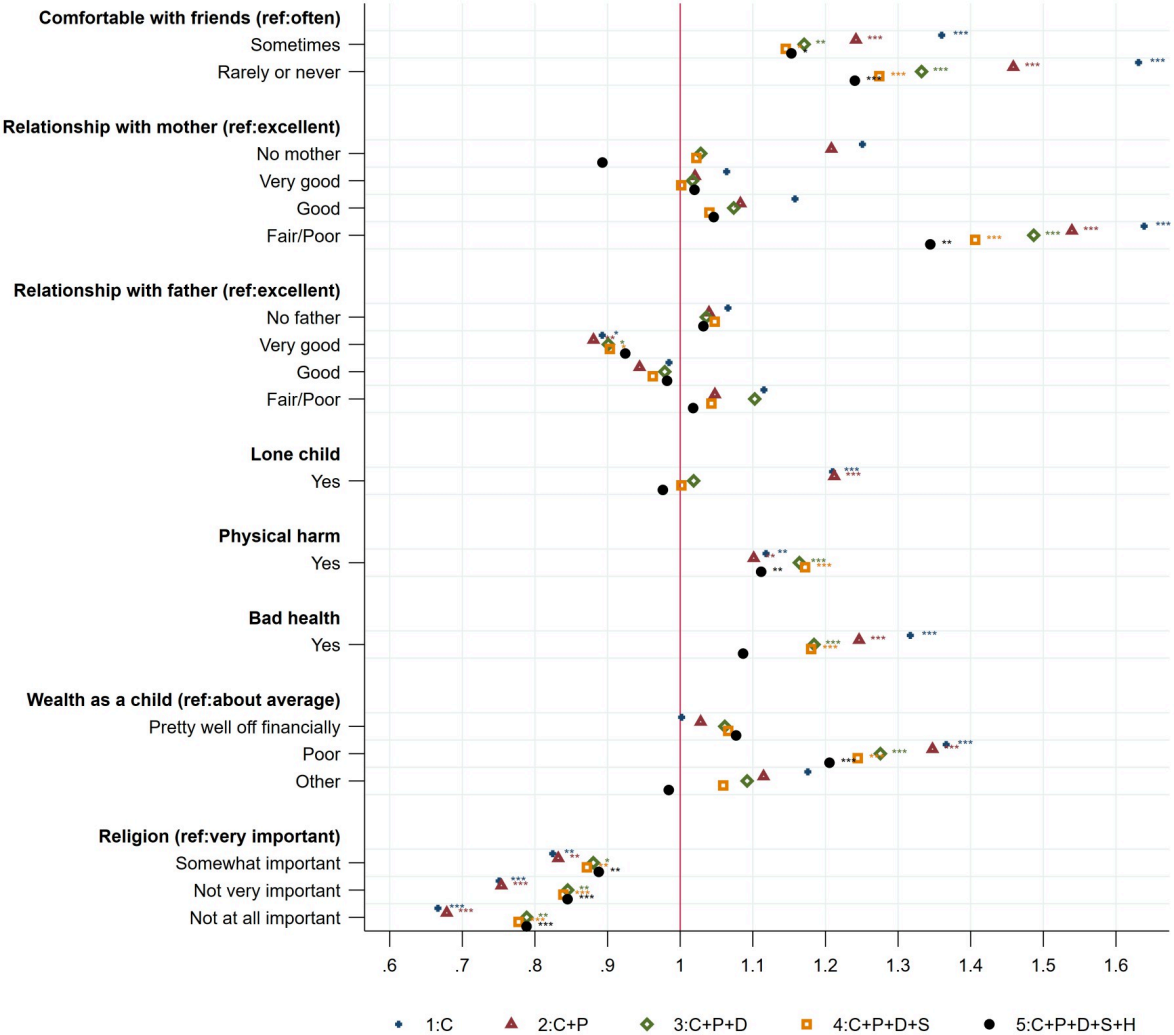
Loneliness at 50+: The role of childhood circumstances. Note: The figure displays odds ratios of the variables describing childhood circumstances for the five different specifications. Specification (1) includes C, a vector of childhood circumstances (wealth; health; quality of the relationship with mother, father, and friends; importance of religion), and I, country-specific fixed effects. Specification (2) includes (1) and P, a vector of the five scores of personality traits (extraversion, agreeableness, conscientiousness, neuroticism, and openness). Specification (3) includes (2) and D, a set of individual socioeconomic and demographic variables at age 50+. Specification (4) includes (3) and S, social support, activities, and network variables at age 50+. Specification (5) includes (4) and H, a vector of individual health information at age 50+. Reading example: in the full specification, reporting having had rarely or never friends with whom spending comfortable time in childhood increases the risk of feeling lonely at age 50+ by 1.24. The pseudo R^2^ and the proportion of correctly specified outcomes are respectively for each specification: (1): 0.0364; 82.89%, (2): 0.0600; 82.99%, (3): 0.0864; 82.96%; (4): 0.1181; 83.39%, (5): 0.1699; 84.09%. * p < 0.05, ** p < 0.01, *** p < 0.001.

First, the association between childhood circumstances and loneliness at age 50+ can be examined. Fig 1 displays the odds ratios of the variables describing childhood circumstances for the five different specifications. This allows for the comparison of the size of the association between childhood circumstances and loneliness at age 50+, after controlling additionally for personality traits, socioeconomic and demographic factors, social support, and health. Having friends with whom they could comfortably spend time as a child is negatively correlated with loneliness in later life. The size of the association decreases gradually with the number of factors included in the model, but it remains significant in all specifications, including in the full model that takes into account the influence of personality traits, health, social support, and socioeconomic and demographic variables. In the full specification (5), the odds of loneliness at age 50+ is 1.15 higher for individuals who *sometimes* had comfortable friends, and 1.24 significantly higher for those who *rarely or never* had comfortable friends, compared to those who replied that they *often* had comfortable friends to with whom to spend time during childhood. In the remainder of this section “Multivariate analysis”, the odds ratios presented are those of the full specification (5).

The quality of the parent–child relationship during childhood is also a significant factor impeding loneliness in later life, especially the mother–child relationship. Those having had a *fair or poor* relationship with their mother as a child have 1.34 higher odds of feeling lonely at age 50+ compared to those with an *excellent* relationship. In contrast, the father–child relationship does not have a significant effect. Similarly, having been the only child in the household during childhood is not a significant factor of loneliness once the later life factors are included (D, S, and H). However, having experienced physical harm as a child increases the likelihood of loneliness at age 50+ by 1.11. The odds of loneliness at age 50+ is 1.09 higher when one had bad health during childhood and 1.21 significantly higher when one grew up in a household with poor wealth. The importance of religion in the household as a child is significantly correlated with feelings of loneliness in later life.

#### Personality traits

**Fig 2:**
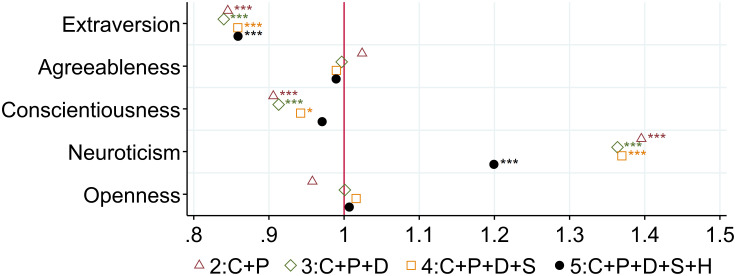
Loneliness at age 50+: The role of personality traits. Note: The figure displays odds ratios of the five scores of personality traits for four different specifications. Specification (2) includes P, a vector of the five scores of personality traits (extraversion, agreeableness, conscientiousness, neuroticism, openness); C, a vector of childhood circumstances (wealth; health; quality of the relationship with mother, father, and friends; importance of religion); and I, country-specific fixed effects. Specification (3) includes (2) and D, a set of individual socioeconomic and demographic variables at age 50+. Specification (4) includes (3) and S, social support, activities, and network variables at age 50+. Specification (5) includes (4) and H, a vector of individual health information at age 50+. Reading example: In the full specification, an increase of 0.5 in the score of neuroticism increases the odds of loneliness by 1.20. The pseudo R^2^ and the proportion of correctly specified outcomes are respectively for each specification: (1): 0.0364; 82.89%, (2): 0.0600; 82.99%, (3): 0.0864; 82.96%; (4): 0.1181; 83.39%, (5): 0.1699; 84.09%. * p < 0.05, ** p < 0.01, *** p < 0.001.

The correlation between personality and loneliness at age 50+ is analyzed by adding the scores of the Big Five personality traits in the model from the second specification. Fig 2 reports the odds ratios of each of the Big Five personality traits for specifications 2, 3, 4, and 5. The size of the association between personality and loneliness at age 50+ remains stable, whatever the specification. An increase of 0.5 in the score for extraversion decreases the odds of feeling lonely by 0.25. The score for conscientiousness is also negatively associated with loneliness, but not significantly in the full specification. Agreeableness and openness seem to be protective personality traits as well, but their effects are not as strong as those of extraversion and conscientiousness. Individuals with a neurotic personality have a higher likelihood of feeling lonely (odds ratio = 1.20).

#### Demographic and socioeconomic factors

**Fig 3:**
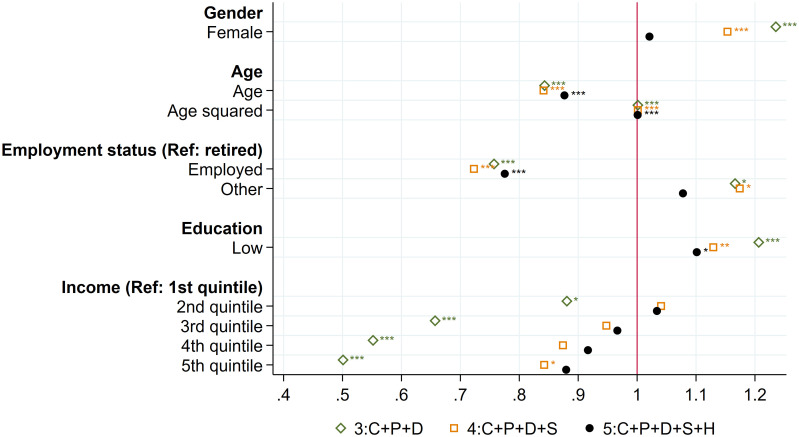
Loneliness at 50+: The role of demographic and socioeconomic factors. Note: The figure displays odds ratios of individual socioeconomic and demographic variables at age 50+ for three different specifications. Specification (3) includes D, a set of individual socioeconomic and demographic variables at age 50+; P, a vector of the five scores of personality traits (extraversion, agreeableness, conscientiousness, neuroticism, openness); C, a vector of childhood circumstances (wealth; health; quality of the relationship with mother, father, and friends; importance of religion), and I, country-specific fixed effects. Specification (4) includes (3) and S, social support, activities, and network variables at age 50+. Specification (5) includes (4) and H, a vector of individual health information at age 50+. Reading example: in the full specification, being employed decreases the odds of loneliness by 0.23. The pseudo R^2^ and the proportion of correctly specified outcomes are respectively for each specification: (1): 0.0364; 82.89%, (2): 0.0600; 82.99%, (3): 0.0864; 82.96%; (4): 0.1181; 83.39%, (5): 0.1699; 84.09%. * p < 0.05, ** p < 0.01, *** p < 0.001.

Fig 3 displays the odds ratios of demographic and socioeconomic variables added in specifications 3, 4, and 5. As expected, the odds of loneliness at age 50+ decreases with income and education. In the full specification, only individuals in the fifth quintiles of income are more likely to feel lonely, and individuals with low education are 1.10 more likely to feel lonely. Being employed is a significant protective factor against feeling lonely in older age, compared to being retired or being a homemaker. When controlling for variable categories C, P, S, socioeconomic factors, and gender, loneliness at age 50+ decreases with age (for individuals aged 50+). Once health factors are included in the model, the significant gender difference in the likelihood of loneliness disappears.

#### Social support

**Fig 4:**
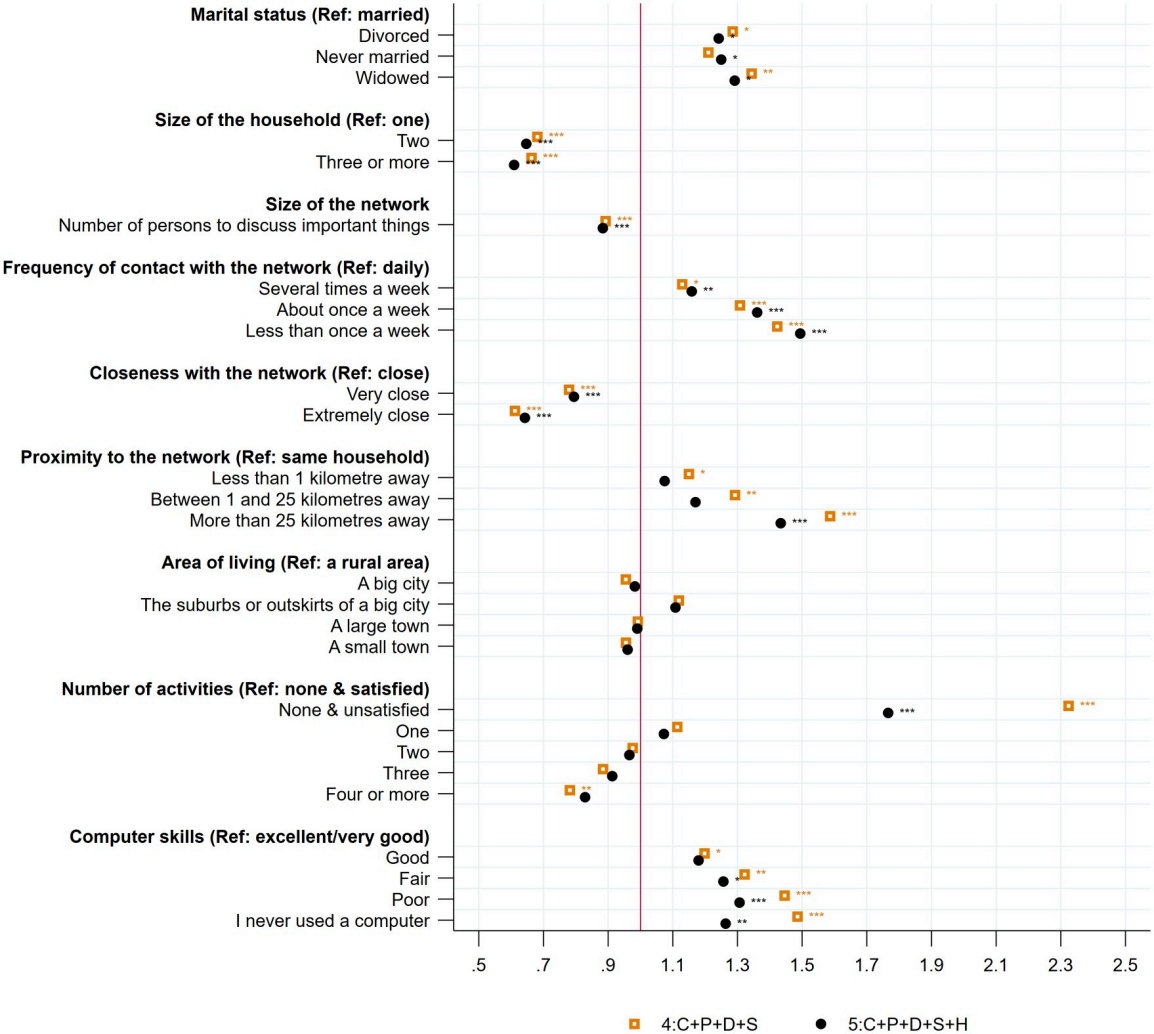
Loneliness at age 50+: The role of social support. Note: The figure displays odds ratios of social support, activities, and network variables at age 50+ for two different specifications. Specification (4) includes S, social support, activities, and network variables at age 50+; D, a set of individual socioeconomic and demographic variables at age 50+; P, a vector of the five scores of personality traits (extraversion, agreeableness, conscientiousness, neuroticism, openness); C, a vector of childhood circumstances (wealth; health; quality of the relationship with mother, father, and friends; importance of religion); and I, country-specific fixed effects. Specification (5) includes (4) and H, a vector of individual health information at age 50+. Reading example: in the full specification, being divorced increases the risk of loneliness by 1.24. The pseudo R^2^ and the proportion of correctly specified outcomes are respectively for each specification: (1): 0.0364; 82.89%, (2): 0.0600; 82.99%, (3): 0.0864; 82.96%; (4): 0.1181; 83.39%, (5): 0.1699; 84.09%. * p < 0.05, ** p < 0.01, *** p < 0.001.

Fig 4 reports the odds ratios of the variables related to the social environment of individuals at age 50+, variables added in specifications 4 and 5 of the model. Having company at home seems to be a protective factor against loneliness. Being widowed, divorced, or never married increases the risk of loneliness compared to being married. Similarly, the risk of loneliness at age 50+ decreases with the size of the household.

Having a network of persons with whom to talk about important things is also an important impeding factor. The risk of loneliness decreases significantly with the size of the network. The reduction in the risk of loneliness also depends on the frequency of contact with the network and the closeness of the network. For instance, the odds of feeling lonely increases by 1.59 when the frequency of contact is less than once a week to never, compared to those with daily contacts. The area of living does not seem to have a significant impact on loneliness at age 50+; however, the geographical proximity of the network is key to reducing the risk of feeling lonely.

Taking part in activities is protective against loneliness for those wishing to participate. Individuals who declared participating in any activity and being unsatisfied with it have an increased odds of loneliness of 1.76. In contrast, taking part in four or more activities significantly reduces the risk of loneliness. Lastly, having sufficient computer skills impedes the odds of loneliness at age 50+. Indeed, individuals who reported having poor computer skills or having never used a computer have increased odds of loneliness of 1.3.

#### Health at age 50+

**Fig 5:**
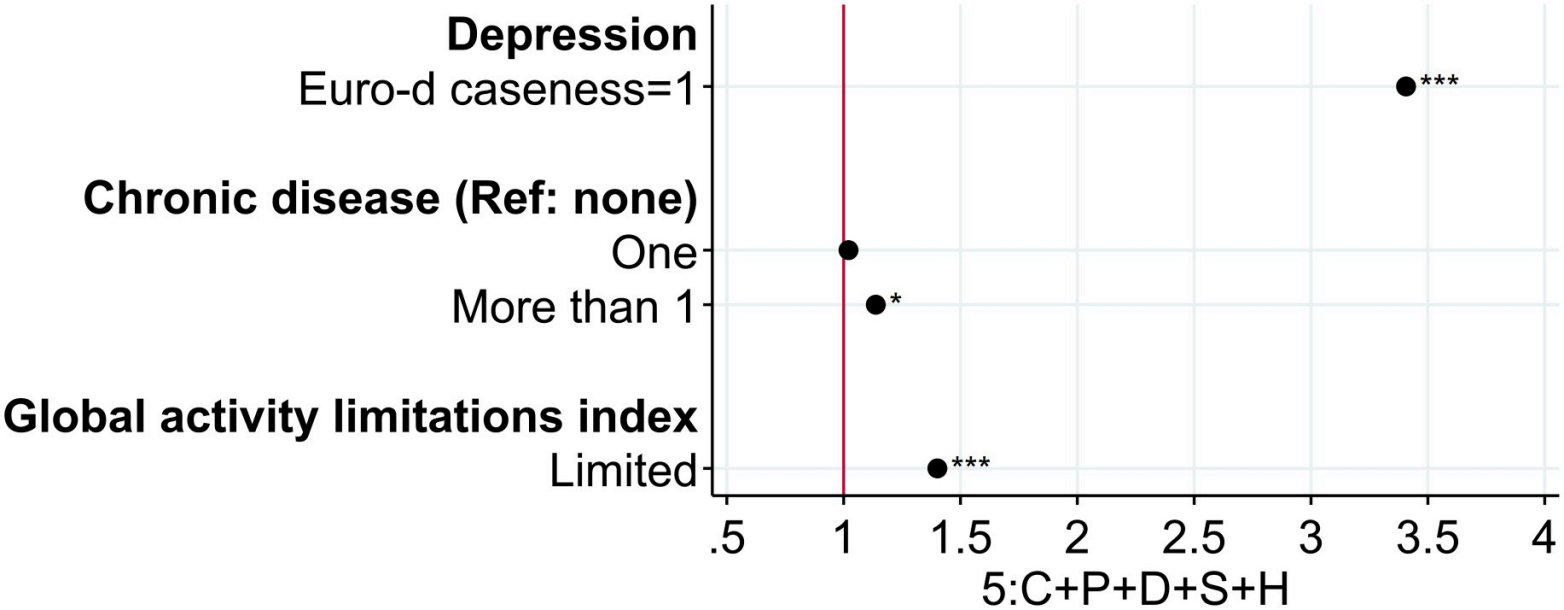
Loneliness at age 50+: The role of health. Note: The figure displays odds ratios of individual health information at age 50+ for the full specification. Specification (5) includes H, a vector of individual health information at age 50+; S, social support, activities, and network variables at age 50+; D, a set of individual socioeconomic and demographic variables at age 50+; P, a vector of the five scores of personality traits (extraversion, agreeableness, conscientiousness, neuroticism, openness); C, a vector of childhood circumstances (wealth; health; quality of the relationship with mother, father, and friends; importance of religion); and I, country-specific fixed effects. Reading example: in the full specification, having limitation with daily activities increases the odds of loneliness by 1.40. The pseudo R^2^ and the proportion of correctly specified outcomes are respectively for each specification: (1): 0.0364; 82.89%, (2): 0.0600; 82.99%, (3): 0.0864; 82.96%; (4): 0.1181; 83.39%, (5): 0.1699; 84.09%. * p < 0.05, ** p < 0.01, *** p < 0.001.

Fig 5 reports the odds ratio of the health variables included in specification 5 of Eq 1. Loneliness at age 50+ significantly decreases with health. The odds are 1.14 higher with more than one chronic disease, 3.41 higher with symptoms of depression, 1.40 higher with limitations with activities.

### The relative importance of the determinants of loneliness at age 50+

To address the final research question of this study, the relative contribution of each category of factors is computed in the variance explained by the model. Table 5 reports the proportions of the explained variance by category of factors with bootstrapped confidence intervals. As expected, in the full specification, ill health status at age 50+ is the main risk factor for loneliness. It contributes to 43.32% of the explained variance, with mental health being the major contributor among the health measures.

**Table 5:** Decomposition of the effects.

	Prop.	S.E.	P-value	Confidence interval	
**Childhood circumstances**	7.50%	0.92%	0.0000	5.70%	9.31%
Relationships	3.73%	0.73%	0.0000	2.29%	5.16%
Health	1.23%	0.46%	0.0070	0.34%	2.13%
Wealth	1.41%	0.45%	0.0020	0.52%	2.31%
Religion	1.13%	0.39%	0.0040	0.37%	1.90%
**Personality traits**	10.42%	1.07%	0.0000	8.33%	12.52%
**Social support**	27.05%	1.62%	0.0000	23.88%	30.22%
Support at home	10.26%	1.04%	0.0000	8.21%	12.31%
Social network	9.87%	0.99%	0.0000	7.94%	11.81%
Activities	4.99%	0.76%	0.0000	3.50%	6.47%
Computer skills	1.93%	0.87%	0.0270	0.22%	3.65%
**Health**	43.32%	1.60%	0.0000	40.19%	46.45%
Chronic diseases	1.82%	0.75%	0.0160	0.34%	3.29%
Mental health	34.93%	1.49%	0.0000	32.01%	37.86%
Limitations	6.57%	0.95%	0.0000	4.71%	8.44%
**Demo & socioeconomics**	6.50%	1.26%	0.0000	4.02%	8.97%
**Country characteristics**	5.20%	0.89%	0.0000	3.45%	6.95%

Note: Proportions of the total variance are displayed by category of factors. Reading example: Personality traits explain 10.42% of the explained variance of the model of loneliness. Bootstrapped confidence intervals are in brackets. The subcategory “Relationships” includes friends, quality of relationship with mother and father, only child at home. “Health” includes bad health as a child and physical harm. “Support at home” includes the marital status and size of the household. “Social network” is composed of size of the network, closeness and proximity of the network, and the area of living. “Activities” includes the number of activities. Mental health is measured with the EURO-D caseness, and limitations with the Global Activity Limitation Index.

Social support in later life is the second highest category of factors and accounts for 27.05% of the explained variance. Social connection at home explains 10.26%. The social network overall explains 9.87% of the model, while engaging in activities and having computer skills in later life account for 6.92% of the explained variance. Life conditions and circumstances during childhood contribute 7.50% and personality traits contribute 10.42%. Demographic and socioeconomic factors account for 6.50% of the explained variance. Country-level characteristics contribute 5.20%.

## Discussion

This paper aimed to study the determinants of loneliness in older adults in Europe; in particular, it investigated whether childhood circumstances are significantly associated with loneliness at older ages. Using data from the SHARE, examining individuals who replied to the SHARELIFE questionnaire in wave 7, an analysis was conducted on loneliness in 17 countries in Europe. The analysis showed significant correlations between life circumstances in childhood and feeling lonely in older age. These correlations remain significant when controlling for personality traits, demographic and socioeconomic characteristics, social support and ill health in later life, and country-specific characteristics. While ill health is the main factor correlated with loneliness at 50+, as expected, the analysis of the relative importance of the determinants reveals that personality traits account for 10.42% of the explained variance and that life circumstances during childhood account for 7.50%. Social support at older ages is the second highest category of factors, accounting for 27.05%—with, interestingly, support at home contributing 10.26%, social network characteristics contributing 9.87% and engaging in activities and computer skills accounting for 6.92% of the explained variance. Demographic and socioeconomic factors account for 6.50% and country-level characteristics contribute 5.20%.

The following limitations must be discussed. One might question how well survey participants remembered their childhood. The SHARELIFE questionnaire uses different techniques to facilitate the recall mechanisms—in particular, an event history calendar with multiple dimensions and visual sequential recollection [88, 97]. These tools have been proven to limit errors in reproducing past life events [98, 99]. For individuals with very high levels of loneliness at 50+, there is a risk that part of the significant association between adverse events in childhood and loneliness in later life might be due to emotional bias. For instance, individuals suffering from depression have been found to recall more accurately and value more intensely adverse past events than healthy individuals [28, 100, 101]. However, given the size of the effect, the association found in this study cannot be explained by this recall bias alone [102]. This study reveals specific types of loneliness, such as situational loneliness, which is due to a specific crisis or loss or to chronic loneliness. For instance, it would be interesting to measure the specific effects of life events at older ages, such as the recent death of a spouse or a divorce, but the number of individuals experiencing these types of events was too low to be introduced in the model. The effects of these events are measured by the marital status factor in the model.

Based on the results and the limitations of this study, the following policy implications can be drawn. First, the findings of this paper confirm the importance of social networks and support in older age. Public interventions aiming at increasing activities and contact among older adults should take into account the different personality traits in adapting the types of activities and interaction possibilities—in particular, for those more prone to suffering from loneliness. This paper also points out the relevance of early life interventions to tackle loneliness in older age. This reaffirms findings from other studies that the quality of relationships at school and at home, as well as adverse life events during childhood, are significant predictors of education, employment, and health in later life [24, 25, 37, 103, 104], though these previous studies did not take into account as many confounding factors in the analysis of the correlation between loneliness and personality as this study did. The importance of personality traits is also confirmed here. The association between personality and loneliness can be explained by a fixed or constant component of personality that remains or changes only marginally over the course of the lifetime, and partly by smaller effects of periods of intense loneliness on personality development [62, 105]. In this view, early interventions and interventions aimed at increasing social support in later life need to be adapted to all personality types [106].

The role of childhood circumstances and the mechanisms explaining the association between loneliness in childhood and loneliness in later life deserve more attention in future research. In light of the trend of increasing childhood loneliness in the past decade [107], as well as the impact of the COVID-19 pandemic on children’s level of loneliness and their expected well-being later in life, this research is now more important than ever.

## Supporting information

S1 Appendix. Sample characteristics (PDF)
