# Supplementary material for: Loneliness among older adults in Europe: The relative importance of early and later life conditions
Source: PLoS One. 2022 May 18;17(5):e0267562. doi: 10.1371/journal.pone.0267562 (PMC9116676; doi:10.1371/journal.pone.0267562)
Supplement: S1 Appendix. Sample characteristics — (PDF) [file pone.0267562.s001.pdf]

S1 Appendix: Sample characteristics

SHARE responses rates in wave 6 and wave 7

The household participation rate is 51.3% and the individual participation rate is 46.8% in wave 6. The household participation rate is 62.8% and the individual participation rate is 56.2% in wave 7 „*Overall, most of the rates are in line with or even above the numbers of comparable surveys in the same period (e.g., European Social Survey 7th edition, 2016)"* P.19 [1]

What is also relevant for this study is the retention rate. It is more than 82% in wave 6-7. "*There is a clear and consistent increase in retention of long-term panel members suggesting a high overall panel stability that is comparable to other studies with even shorter time intervals between interviews*." P34 and "*Retention stabilizes after few waves at a very high level indicating that the survey succeeds in keeping respondents participating over a remarkable long time despite their, on average, advanced age*." P.39 [1]

Work sample

The work sample is composed of individuals eligible to the social network module in wave 6, and to the SHARELIFE history questionnaire in wave 7. The number of observations amounts 34,370. Individuals not eligible for the social network module are those who participated in SHARE for the first time in wave 6 or because they were respondents from countries that did not participate in wave 4 and were thus eligible to receive the SN in wave 6, but had a proxy interview (sn014 = 5), making them ineligible. For more information see [2].

For the analysis, individuals aged below 50, with no answer to the questions related to the loneliness scale, and from Poland (too few observations, 29) were excluded. Therefore the sample includes 33,523 observations (see Table S1).

Table S1. Exclusion criteria.

**Criteria**	**N**
Participated in wave 6	68,186
Participated in wave 6-7	52,570
Replied to the SHARELIFE life history interview	39,368
Eligible for social networks module (sn)	34,370
Below 50	33,953
Missing loneliness scale (3.64%)	33,606
Country Poland	33,523

Among these 33,523 observations a small proportion of missing values has been imputed by the SHARE data project team using multiple imputation techniques (hot-deck method and fully conditional specification (FCS) method). The hot-deck method is used for various types of variables affected by negligible fractions of missing values (usually, much less than 5 percent) and the FCS method for monetary variables affected by more relevant fractions of missing data (See detailed description of the methods and the procedures in [3, 4]. Table S2 displays the proportion of imputed observations by variable. The rate of imputed observations is very low for all the variables used in the analysis except income. Therefore, robustness checks have been performed, see below.

Some variables have not been imputed and include missing observations (non-responses). Table S3 reports the proportion of missing observations in the variables used. To assess whether missing observations impact the results, the model was run with the missing observations and was concluded that the estimates are very similar to the main specification and that the missing observations are never statistically significant. To check whether the imputed observations in the income variable change the results, the model was also run excluding the individuals with imputed income in addition to the missing observations. All three specifications lead to similar estimates.

Table S2. Proportion of imputed observations by variable.

Satisfaction with no activity	1.14%
Number of activity	0.23%
Mobility	0.00% (1 observation)
Current job situation	0.04%
Marital status	0.43%
Education	1%
Depression scale	0.55%
Limitations with activity	0.01%
Number of chronic diseases	0.02%
Income	34.98%
N=	33,523

Table S3 Proportion of missing observations by variable.

Friends comfortable spending time with	1.52%
No other child in household	0.59%
Physical harm	3.98%
Bad health during childhood	0.63%
Wealth during childhood	4.02%
Religion	1.34%
Frequency of contact with the network	2.60%
Closeness to the network	2.58%
Proximity to the network	6.06%
Computer skills	0.04%
Area of living	4.81%
N=	33,523

References:

1. Bergmann M, Kneip T, De Luca G, Scherpenzeel A. Survey participation in the Survey of Health, Ageing and Retirement in Europe (SHARE), Wave 1-7. Based on Release 7.0.0. . Munich: SHARE-ERIC.; 2019.

2. Malter F, Borsch-Supan A. SHARE Wave 6: Panel innovations and collecting Dried Blood Spots. Munich: MEA, Max Planck Institute for Social Law and Social Policy; 2017.

3. De Luca G, Celidoni M, Trevisan E. Item nonresponse and imputation strategies in SHARE Wave 5. Munich: : Munich Center for the Economics of Aging (MEA) at the Max Planck Institute for Social Law and Social Policy (MPISOC). 2015.

4. Bergmann M, Scherpenzeel A, Börsch-Supan A. SHARE Wave 7 Methodology: Panel Innovations and Life Histories. Munich; 2019.
